# Impact of in vitro fertilization state mandates for third party insurance coverage in the United States: a review and critical assessment

**DOI:** 10.1186/s12958-022-00984-5

**Published:** 2022-08-04

**Authors:** Benjamin J. Peipert, Melissa N. Montoya, Bronwyn S. Bedrick, David B. Seifer, Tarun Jain

**Affiliations:** 1 Department of Obstetrics and Gynecology, Duke University School of Medicine, Duke University Hospital, 2301 Erwin Rd, 27705 Durham, NC USA; 2 Department of Gynecology and Obstetrics, The Johns Hopkins University School of Medicine, Baltimore, MD USA; 3 Division of Reproductive Endocrinology and Infertility, Department of Obstetrics, Gynecology and Reproductive Sciences, Yale University School of Medicine, New Haven, Connecticut, USA; 4 Division of Reproductive Endocrinology and Infertility, Department of Obstetrics and Gynecology, Northwestern University Feinberg School of Medicine, Chicago, IL USA

**Keywords:** Infertility, Assisted reproductive technology, In vitro fertilization, Insurance mandates, Health policy

## Abstract

The American Society for Reproductive Medicine estimates that fewer than a quarter of infertile couples have sufficient access to infertility care. Insurers in the United States (US) have long considered infertility to be a socially constructed condition, and thus in-vitro fertilization (IVF) an elective intervention. As a result, IVF is cost prohibitive for many patients in the US. State infertility insurance mandates are a crucial mechanism for expanding access to fertility care in the US in the absence of federal legislation. The first state insurance mandate for third party coverage of infertility services was passed by West Virginia in 1977, and Maryland passed the country’s first IVF mandate in 1985. To date, twenty states have passed legislation requiring insurers to cover or offer coverage for the diagnosis and treatment of infertility. Ten states currently have “comprehensive” IVF mandates, meaning they require third party coverage for IVF with minimal restrictions to patient eligibility, exemptions, and lifetime limits. Several studies analyzing the impact of infertility and IVF mandates have been published in the past 20 years. In this review, we characterize and contextualize the existing evidence of the impact of state insurance mandates on access to infertility treatment, IVF practice patterns, and reproductive outcomes. Furthermore, we summarize the arguments in favor of insurance coverage for infertility care and assess the limitations of state insurance mandates as a strategy for increasing access to infertility treatment. State mandates play a key role in the promotion of evidence-based practices and represent an essential and impactful strategy for the advancement of gender equality and reproductive rights.

## Background

Infertility is defined as the failure to achieve successful pregnancy after at least 12 months of regular unprotected intercourse in women younger than age 35, or within 6 months in women 35 or older [1, 2]. It represents one of the most common diseases among reproductive-age individuals, affecting approximately 15% of all couples in the United States (US) [3]. Common treatments for infertility include ovulation induction (OI), ovarian stimulation (OS) in combination with timed intercourse or intrauterine insemination (IUI), and assisted reproductive technologies (ART), which include all treatments involving the manipulation of eggs or embryos. In vitro fertilization (IVF) currently represents the most effective form of ART and overall treatment for infertility. Between 1995 and 2018, the IVF live birth rate per initiated cycle among women younger than 35 years old increased from 25 to 52% [4, 5], whereas non-IVF fertility treatments have remained associated with relatively low live birth rates [5–7].

Over 9 million American women had ever received infertility services in 2018. The total number of IVF cycles performed in the US has increased 153x in the past 33 years to over 300,000 cycles per year, and in 2018 IVF accounted for 2% of infants born in the US [5, 8]. Still, the American Society for Reproductive Medicine (ASRM) estimates that only 24% of infertile couples are able to access the full extent of services necessary to become pregnant [9]. Infertility was formally defined as a disease by the World Health Organization in 2009 [10], and reproduction is considered a “fundamental interest and human right” by ASRM [11]. Insurers in the US historically considered infertility to be a socially constructed affliction, and thus IVF to be an elective intervention [12, 13]. As such, IVF has primarily been paid for out-of-pocket (OOP).

The present and historical lack of insurance coverage for infertility implies that the condition is undeserving of financial support and minimizes the suffering of patients with infertility, despite the profound medical, psychologic, social, and economic harms associated with the condition [14]. The cost of IVF remains the greatest barrier to infertility care in the US [15–18]. ASRM states that the average cost of an IVF cycle in the US is $12,400 [19]. However, other studies estimate the cost per cycle at approximately $20,000-$25,000, and the cost per live birth can exceed $60,000 in some of the most expensive parts of the country [20, 21]. Studies have shown that an IVF cycle in the US costs 271% more than the mean cost in 25 other countries [22]. Given the extraordinary financial burden of infertility, insurance coverage for infertility treatment has been heavily debated at both the state and federal level [23–28].

State infertility insurance mandates are a crucial mechanism for expanding access to fertility care in the US. The first state insurance mandate for third party coverage of infertility services was passed by West Virginia in 1977 as part of a series of regulatory bills governing health-maintenance organizations (HMOs) [29]. However, the original mandate makes no mention of coverage for specific infertility treatments, in part due to the fact that the law predates the first successful IVF birth in 1978 [30]. In the 1980s, advocates began lobbying for state legislatures to mandate private health insurance companies to cover the cost of infertility treatment, and in 1985, Maryland passed the country’s first mandate requiring third party payers to cover costs associated with IVF [31]. To date, 20 states have passed legislation requiring insurers to cover or offer coverage for the diagnosis and treatment of infertility, with 13 states mandating third party coverage for IVF [32, 33]. The resulting number of births from IVF occurring in states with mandated infertility coverage has increased from < 1% in 1985 to over 50% in 2018 [5].

**Fig. 1:**
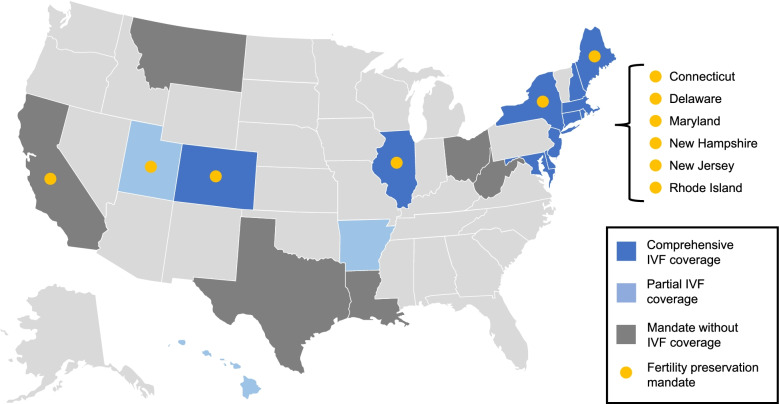
Summary of infertility and fertility preservation mandates in the United States

Discussions specific to state insurance mandates for third party coverage of infertility have been detailed at length in recent publications [32, 34]. A brief overview of current infertility mandates in the US is shown in Fig. 1; Table 1 [33]. The current 20 state infertility mandates for third party coverage are extremely heterogeneous with a wide range of patient eligibility requirements, covered services, restrictions, and exclusions. Twelve mandates (60%) require coverage for fertility preservation (FP) for patients at risk of iatrogenic infertility, especially cancer patients. Eight mandates (40%) require patients to demonstrate infertility for a duration longer than the medical definition in order for their infertility treatments to qualify for coverage. Six out of thirteen states (46%) with a mandate to cover IVF require patients to first attempt less costly treatments for infertility. Eleven states (55%) place a lifetime limit on the number of cycles or total dollar amount of infertility care that can be provided under the infertility mandate. Three states (15%) have age restrictions (ages 42–46) after which mandated benefits are no longer covered. Religious and small employer exemptions are common, appearing in 8 (40%) and 6 (30%) states, respectively. Arkansas, Hawaii, and Texas require a patient’s eggs to be fertilized with their husband’s sperm to qualify for coverage under the mandate, thus discriminating against couples who do not conform to standards of heteronormativity and traditional gender roles, in addition to couples with azoospermia or severe male factor.

**Table 1:** Summary of state infertility insurance mandates in the United States

State	Mandated services		Eligibility criteria		Additional requirements		Exemptions	
	IVF	FP	Years of infertility^a^	IVF age limit (years)	Requires use of spouse’s sperm	Benefits limit	Small Employers	Religious
Arkansas	X		2		X	X		
California		X	1					X
Colorado	X	X	0.5–1			X		X
Connecticut	X	X	1			X		X
Delaware	X	X		Egg retrieval < 45 Transfer < 50		X	X	X
Hawaii	X		5		X	X		
Illinois	X	X	1			X		X
Louisiana								
Maine^b^	X	X						
Maryland	X	X	1			X	X	X
Massachusetts	X		0.5–1					
Montana								
New Hampshire	X	X						
New Jersey	X	X	0.5–1	< 46		X	X	X
New York	X	X	0.5–1			X	X	
Ohio								
Rhode Island	X	X	1	25–42			X	
Texas	Mandate to offer		5		X	X		X
Utah	Partial^c^		Qualifying genetic conditions^d^					
West Virginia								

^a^In absence of a qualifying condition, such as: (DES) exposure; blocked or surgically removed fallopian tubes that are not the result of voluntary sterilization; male factor infertility

^b^Effective January 1, 2023

^c^Utah’s mandate includes $4,000 toward infertility treatments as part of optional maternity benefits

^d^Following a waiver application process, Utah’s mandate includes IVF and genetic testing for certain genetic traits associated with qualifying conditions for Medicaid and patients and the Public Employees’ Health

Several studies analyzing the impact of infertility mandates have been published since the early 2000s. In general, these studies refer to a “comprehensive” or “complete” IVF mandate as one that requires insurance companies to provide coverage for the cost of IVF with minimal restrictions to patient eligibility, plan exemptions, and lifetime limits to benefits received [14, 22, 35–39]. While prior analyses have varied in the exact states meeting the definition of a comprehensive IVF mandate, there is general agreement that this group of states mandates (currently consisting of Connecticut, Colorado, Delaware, Illinois, Maine, Massachusetts, Maryland, New Jersey, New York, and Rhode Island) significantly reduce financial barriers to IVF.

In this review, we characterize and contextualize the existing evidence of the impact of comprehensive IVF state insurance mandates upon access to care, practice patterns, and reproductive outcomes. An overview of contemporary data on these endpoints is summarized in Table 2. Furthermore, we summarize the arguments in favor of insurance coverage for IVF and assess the limitations of state insurance mandates as a strategy for increasing access to care in the treatment of infertility.

**Table 2:** Overview of contemporary data on the impact of comprehensive / mandated IVF insurance coverage

Variable	Comprehensive / Mandated IVF coverage	Non-comprehensive / Non-mandated	*P*-value	Data Year	Source
Utilization					
Per capita IVF utilization^a^	6.2 per 1,000 women ages 25–44	2.7 per 1,000 women ages 25–44	*p* < .001	2018	Peipert et al. 2022 [39]
Practice Patterns					
Embryos per transfer^a^	1.30	1.36	*p* < .001	2018	Peipert et al. 2022 [39]
Frozen embryo transfer	66.1%	76.3%	*p* <. 001	2018	Peipert et al. 2022 [39]
ICSI utilizaiton^b^	62.5%	67.6%	*p* =. 06	2016	Zagadailov et al. 2020 [40]
PGT utilization^c^	19.6%	27.6%	*p* < .001	2014–2016	Bedrick et al. 2022 [41]
Outcomes					
Live birth rate^a^	35.4%	33.4%	*p* < .001	2018	Peipert et al. 2022 [39]
Multiple birth rate^a^	10.2%	13.8%	*p* < .001	2018	Peipert et al. 2022 [39]

^a^Includes all nondonor cycles reported to the CDC in 2018; comprehensive group included Connecticut, Illinois, Massachusetts, Maryland, New Jersey, and Rhode Island

^b^Among fresh nondonor cycles; Arkansas and Hawaii additionally included in comprehensive group

^c^Among non-banking cycles; Arkansas, Hawaii, Montana, Ohio, and West Virginia additionally included in mandate group

## Main text

### Access to fertility treatments

#### Utilization of fertility treatments

Studies on the impact of infertility state mandates have consistently demonstrated a higher per capita utilization of IVF in states with comprehensive insurance coverage for the procedure. A landmark study published in the New England Journal of Medicine by Jain et al. in 2002 demonstrated that complete insurance coverage for IVF was associated with a utilization rate 277% higher than in states without coverage [22]. Later studies from the same period reaffirmed this association [37]. Early research leveraged data collection pre- and post-enactment of state mandates to measure the temporal impact of infertility mandates. For example, a study on the impact of the New Jersey (2001) and Connecticut (2005) mandates found that ART use increased more in these states following passage of infertility mandates compared to non-mandated states between 1996 and 2013 [42]. A recent analysis using data from the 2018 Centers for Disease Control and Prevention (CDC) ART Fertility Clinic Success Rates Report confirmed that comprehensive IVF insurance mandates are associated with more than double the rate of IVF cycle utilization per capita (6.2 vs. 2.7 cycles per 1,000 women ages 25–44) than states without mandates, even after age-standardization and age-stratified sub-analysis [39].

Modeling of markets for infertility treatment similarly demonstrates higher utilization of IVF in states with insurance mandates [43]. For every 1% drop in the cost of an ART cycle in terms of disposable income, utilization increases by 3.2% [44]. The increase in IVF utilization likely results from two synergistic mechanisms. As cost per cycle decreases, demand for IVF increases, leading to both the opening of new clinics and an increased capacity of existing clinics. CDC data from 2018 supports this hypothesis: the number of fertility clinics among 6 states with comprehensive IVF insurance mandates (Connecticut, Illinois, Massachusetts, Maryland, New Jersey, and Rhode Island) was 1.31 per 100,000 women ages 25–44, compared to 1.00 per 100,000 in states without these mandates. Likewise, cycle volume per clinic was 80% higher in states with comprehensive mandates compared to non-comprehensive states (478 vs. 267 cycles per clinic per year, respectively) [39].

Although state mandates lead to a substantial increase in IVF utilization, significant barriers in access to care persist. Per capita utilization of ART in the US lags behind that of other developed countries whose national health programs provide public funding for IVF. In prior studies, ART accounts for 1.5% of births in the US compared to 3% in Europe, with an even higher percentage among countries with full or partial coverage for IVF, including Belgium, Denmark, and Sweden (3.5–5.9%) [14, 45, 46]. According to an analysis from the European Society of Human Reproduction and Embryology (ESHRE), the estimated demand for ART in a given population is ≥ 1,500 cycles per million people per year [47]. In 2013, the US met only 40% of the presumptive national demand for ART, compared to 62% in UK, ≥ 100% in Scandinavia, and ≥ 100% in Australia [48, 49].

Historically, opponents to mandated coverage of fertility treatments have argued that the aggregate cost of increased per capita IVF utilization and the resulting increase in associated multiple births are too expensive to be covered by insurance [32]. On the contrary, numerous studies have demonstrated that the per capital incremental cost of fertility coverage is minimal and significantly less than many routinely covered treatments [32, 50–53]. A 2006 survey of over 600 US employers who offered an infertility benefit found that > 90% reported that their infertility benefit did not add significant costs [54]. Other studies among large employers indicate that a limited infertility benefit accounts for < 0.5–0.8% of total health care expenditures [55, 56], and more recent data from Massachusetts showed that infertility treatment accounted for 0.12–0.95% of total premium costs [14]. Furthermore, some economists argue that the cost of additional IVF cycles pale in comparison to the socioeconomic advantages of population growth, especially for countries with negative or flat population growth and/or an aging population [57]. American society in particular is dependent on population growth to sustain economic growth and support social programs, such as Social Security [58]. If the existing demand for ART in the US is to be met, expanded insurance coverage and technologic advances to improve the affordability of IVF are essential.

#### Disparities in access to care

Racial and ethnic disparities in access to fertility care are well documented. Based on the National Survey for Family Growth 2006–2010, non-Hispanic Black women were > 30% more likely to report a 12-month history of infertility compared to non-Hispanic white women [59] but > 40% less likely to report ever-use of fertility services [60]. A similar discrepancy has been described among Hispanic women, although of a lesser magnitude [17]. Even among those patients who access ART, poorer outcomes have been observed for patients of racial and ethnic minorities. Multiple studies have demonstrated that black, Hispanic, and Asian women have lower clinical pregnancy and live birth rates compared to their white counterparts [61–63]. This may be in part due to delays in presentation to medical care as black women who eventually present for fertility care do so on average 1 year later than their white counterparts [64]. Race has been consistently shown to be an independent predictor of IVF outcome [65–68]. It has been theorized that these poorer outcomes following fertility treatments among black women could be due to increased rates of obesity, tubal factor infertility, and fibroids [28, 62, 69]. However, these disparities in ART outcomes are almost certainly influenced by sociocultural barriers encountered by patients of racial and ethnic minorities, such as stigma against infertility and fear of disappointing a partner [70–73]. Further studies are needed to better characterize the intersectional etiologies of racial and ethnic disparities in IVF access and outcomes.

Additional inequities in accessing ART have been characterized based on education, geography, and income. One study by Bitler and Schmidt found that individuals with a high school education or less were significantly more likely to report infertility than individuals with a four-year college degree; however, IVF utilization was strongly correlated with higher income and a greater concentration of reproductive-age women with a bachelor’s degree in a given area [17]. Differential access to IVF has also been directly attributed to a greater number of physicians per-capita performing IVF in areas with higher income levels [12]. Furthermore, it is estimated that almost 25% of the US population lives in an area without access to ART, predominantly in rural areas [74].

Even in states with mandates, infertility care is disproportionately used by college-educated, high socioeconomic status non-Hispanic white women. In 2005, an extensive survey study was conducted among individuals accessing infertility services in Massachusetts, a state with mandated insurance coverage for the diagnosis and management of infertility, including IVF [16]. Despite the mandate, Hispanic/Latina women were significantly underrepresented among patients accessing fertility services; African American women were similarly under-represented, but this finding did not reach statistical significance. None of the survey respondents had less than a high school education, compared to 15.1% in the state population, and nearly 50% of respondents had an advanced degree, compared to 12.4% in the state population. 60% of respondents had an annual household income over $100,000 per year, compared with 17.7% in the state at large. A 2006 study similarly found that older, more educated women exhibit an increase in utilization as a result of fertility mandates [75].

More recent studies on the impact on newer mandates on access to ART have been mixed. Analyses on the impact of the Connecticut and New Jersey mandates found that there was a significant increase in embryo transfers among non-Hispanic blacks and Hispanics in Connecticut, but not in New Jersey [42]. A 2017 study using national data from the CDC’s NASS reporting system [76] saw ART utilization rise disproportionately in states with IVF insurance mandates compared to states without such mandates, regardless of race/ethnicity. However, IVF mandates did not correct the existing disparities in ART utilization among black non-Hispanic and Hispanic patients, consistent with multiple other studies [64, 76, 77]. Similarly, the impact of mandates on live birth rate differs by race and ethnicity [17]. Mandates have been shown to increase birth rates for white women ages 35 and older, with no statistical difference found for birth rates among black women, regardless of age [17].

### Practice patterns

#### Elective single embryo transfer (eSET)

In 2011, fertility treatments contributed to 36% of all twin births and 77% of all triplet and higher order multiple births [78]. Ability to prevent iatrogenic multiple births is higher with ART relative to non-ART fertility treatments (e.g., ovulation induction/IUI), as elective single embryo (eSET) transfer virtually eliminates the chance for multiple gestation [79]. However, financial barriers often incentivize patients to first attempt conception with less effective fertility treatments with higher risk for multiples. When using IVF, the same financial barriers may influence the transferring of more than a single embryo, raising the risks of multiple pregnancy and its concomitant associated morbidity [40].

Numerous studies have shown that state IVF mandates reduce the number of embryos per transfer, and the magnitude of this impact has increased over time [22, 35, 36, 42, 80]. In the 2002 Jain et al. study, the number of fresh embryos transferred per cycle was lower in states requiring complete insurance coverage (3.25) than in states requiring partial (3.54) or no coverage (3.59) [22]. A study published this year using similar methods to the Jain study demonstrated that states with comprehensive IVF mandates transferred 1.30 embryos per transfer, compared to 1.36 embryos per transfer in states without these mandates [39]. The magnitude of this difference was likely diminished by the finding that fresh embryo transfer was more common in states with comprehensive mandates and more embryos per transfer were noted in fresh (1.55 and 1.67 embryos per transfer in comprehensive and non-comprehensive states, respectively) compared to frozen embryo transfers (1.18 and 1.27 embryos per transfer, respectively) [39]. It is possible that physicians feel pressure to succeed on the first attempt due to high costs to patients when IVF is paid for OOP rather than through insurance, resulting in a higher number of embryos transferred per cycle [22, 81, 82]. A large modeling study using international ART affordability data showed that ART affordability is independently associated with the number of embryos transferred per cycle: a decrease in the cost of a cycle by 10% of disposable income results in an approximately 5% increase in single-embryo transfer rate and a 7.5% decrease in the percentage of fresh cycles transferring 3 or more embryos [44]. In general, the higher utilization of eSET in states with IVF mandates tends to be driven by differences in utilization among younger patients [36]. This is consistent with current ASRM guidelines which promote single embryo transfer of euploid cleavage-stage embryos and blastocysts for most patients, but permit the transfer of up to 3 non-euploid blastocysts and up to 5 non- euploid, non-favorable cleavage-stage embryos for women ages 41–42 [83–85].

#### Intracytoplasmic sperm injection (ICSI)

Between 1995 and 2004, the percentage of IVF cycles using ICSI increased dramatically, from 11% to over 57% of cycles [86]. This may be attributable to the finding that many fertility centers expanded the indications for ICSI beyond male factor infertility during this time, including fertilization failure in a prior IVF cycle, poor-quality of few oocytes, previously cryopreserved oocytes, use of preimplantation genetic testing (PGT), and in HIV or HCV discordant couples [84]. However, there is limited proven benefit of ICSI in improving outcomes compared to conventional IVF in the absence of male factor infertility [84, 87]. ASRM published a committee opinion in 2020 stating that ICSI has not been associated with improved live birth rates for unexplained infertility or advanced maternal age (AMA), and no studies have addressed whether ICSI improves outcomes for poor-quality oocytes [87]. As a result, the use of ICSI for cases without male factor infertility or a history of prior fertilization failure is not supported by currently available evidence [87].

The evidence on the impact of infertility mandates on ICSI utilization remains mixed. Early studies on the impact of the Connecticut and New Jersey mandate showed that ICSI use per cycle did not significantly differ from non-mandated states [42]. More recent analyses by Provost et al., Dieke et al. and Zagadailov et al. showed that cycles in mandate states were less likely to use ICSI compared to nonmandated sates [38, 40, 88, 89]; specifically among non-male factor infertility cycles, growth in ICSI use was greater among non-mandated states compared to mandated states [88]. More research is needed to understand how infertility mandates could lead to lower ICSI utilization among non-male factor cycles. Mandates may reduce the pressure on patients and providers to utilize additional interventions to reduce the chance of fertilization failure [44, 88]. Furthermore, insurance companies may be restricting ICSI use outside of cases with demonstrated benefit compared to conventional IVF.

#### Preimplantation genetic testing (PGT)

The use of PGT has risen dramatically in the last three decades [90], driven largely by its use for aneuploidy (PGT-A) [41]. According to the CDC, in 2018 37.7% of all IVF cycles used PGT [5]. Like ICSI, PGT is an IVF “add-on” that may be covered by insurance in certain circumstances (i.e., known genetic mutation) but is often paid for OOP. Despite growing evidence that PGT-A does not improve chance of livebirth in all populations [91–93], providers and patients may feel pressured to use PGT-A to maximize chance of livebirth and clinic livebirth rates [93]. In their study of non-banking IVF cycles reported to SART between 2014 and 2016, Bedrick et al., found that PGT use for all indications was 31% less likely to be performed in states with an insurance mandate [41]. There was no difference in use of PGT for single genetic mutations (PGT-M) between states with and without insurance mandates. However, states without an insurance mandate had significantly higher use of PGT-A and PGT for elective sex selection. The authors postulated that patients in states without an insurance mandate may have more disposable income to pay for IVF add-ons, such as PGT for elective sex selection. Alternatively, patients may feel compelled to maximize the perceived chance of livebirth per cycle versus paying for an additional cycle OOP.

#### Fresh versus frozen embryo transfer

Recent trials have not demonstrated a difference in live birth rates between fresh and frozen embryo transfers [94, 95]. However, two studies have found that fresh embryo transfers are relatively more common in Europe [45] and in states with comprehensive IVF insurance mandates [39]. The mechanism behind these findings remains somewhat unclear. Many state infertility insurance mandates in the US do not cover the costs associated with freezing or storing embryos, either implicitly or explicitly (as in the case of New Jersey) [32, 33, 96]. According to Fertility IQ, frozen embryo storage costs approximately $2,000 for 5 years of storage [97]. While this figure pales in comparison to the total cost of an IVF cycle, the OOP cost of storing embryos for future frozen embryo transfer may be greater than the cost of additional IVF cycle with fresh embryo transfer under insurance coverage. Additional research is needed to determine how differences in OOP spending could incentivize differential embryo transfer practices.

### Multiple & live birth rates

#### Multiple birth rates

In 2012, over a quarter of all live births resulting from ART transfer cycles were multiple births, 97% of which were twins and 3% of which were triplets or higher order births [79]. Fertility treatments account for approximately one-third of all twin births and three-quarters of triplet and higher-order births [78]. Early studies on changes in eSET utilization associated with mandates showed a promising trend toward reducing the risk of multiple births. Following the passage of the Illinois, Massachusetts, and Rhode Island infertility mandates, the rate of transfer of 3 or more embryos dropped significantly in Massachusetts and Rhode Island compared to non-mandated states; however, this did not result in a significant decrease in the rate of twins, and only Massachusetts demonstrated a lower risk of triplet or higher order gestations [80]. More recent studies have demonstrated an association between increased eSET utilization, insurance mandates, and decreased rates of multiple births. A 2016 analysis by Provost et al. found that the rate of SET was significantly higher in mandated states among women younger than 35 years with a day 5 transfer (21.8% vs. 13.1%), and multiple birth rates were significantly lower in states with mandates (29.0% vs. 32.8%, OR 0.87 [0.80–0.94]); however, when examining by age stratification, this association only remained statistically significant in women younger than 35 years old who underwent transfer on day 5 (33.1% vs. 38.6%, OR 0.81 [0.71–0.92]), and eSET was rarely seen among women older than 37 years. The most recent study on multiple birth rates found that states with comprehensive IVF mandates had a multiple birth rate of 10.2% compared to 13.8% in states without these mandates [39].

Of note, the resulting increase in utilization of IVF following the passage of mandates often outweighs the increase in elective single embryo transfer. Mandates reduce the number of embryos transferred per cycle, but also reduce the financial burden of ART such that existing patients can increase the number of attempted cycles and invite in a new group of patients who previously did not have access to ART due to prohibitive costs. As such, states with insurance mandates requiring ART coverage ultimately tend to have higher ART-related multiple births than states without mandates [46].

#### Live birth rates

Several early studies demonstrated that state infertility mandates result in a deleterious effect on overall live birth rates. The percentage of cycles resulting in pregnancy and live births, oocyte retrievals resulting in live birth, and embryo transfers resulting in live birth are significantly lower in states with mandate coverage for IVF [22]. A 2003 study by Reynolds et al. found that 3 states with mandates had lower live birth rates compared to states without mandates (24%, 34%, and 35% vs. 40%) [80]. A later study from 2008 found that comprehensive mandates were associated with a small yet significant reduction of 0.04 births per cycle [37].

Several theories attempt to explain the lower pregnancy rates per cycles observed in states with insurance mandates. With many insurers incentivizing or requiring eSET, a lower number of embryos are often transferred per cycle in states with mandates. Furthermore, mandated states tend to have a larger proportion of older women with lower likelihood of success as a percentage of all patients undergoing IVF [22]. However, the increase in utilization among women ages 38–45 in mandated states is only slightly higher than the utilization among women ages 25–37 (293% vs. 269% increase in fresh-embryo cycles, 211% vs. 201% increase in frozen embryo transfers) [22]. A 2010 study by Banks et al. demonstrated a significantly lower live birth rate in states with mandates, even among women under age 35 [35]. Therefore, the difference in proportion of older patients is unlikely to account for the large discrepancy in pregnancy rates between states requiring coverage and those that do not.

Independent of age, patient selection factors may result in lower pregnancy and live birth rates in states with IVF mandates. Insurance companies often require women undergo a certain number of cycles with OI/IUI prior to IVF. Since this is not a requirement in states that do not require insurance coverage of IVF, it is possible that women in non-mandated states more quickly proceed to IVF to save money, resulting in a higher proportion of patients with a favorable prognosis given increased success rates with this procedure as compared to other fertility treatments.

A handful of more recent studies from the 2010s have demonstrated a more favorable relationship between IVF mandates and live birth rates. Between 2000 and 2016, live birth rates were higher among non-mandates states, but a sub-analysis of cycles from 2012 to 2016 showed no difference in live birth rate per cycle between mandated and non-mandated states [40]. Two 2016 studies by Provost and Mancuso et al. showed state mandates resulted in significantly lower rates of multiple births without a significant impact on clinic-level live birth rates [38, 98]. A 2017 by Jungheim et al. which stratified patients based on insurance coverage for IVF noted that while insurance coverage was not associated with the probability of live birth in individual cycles, it was associated with a statistically significant increase in the cumulative live birth rate after 4 cycles (58.5% vs. 50.5%, *p* = .001) [99]. The most recent analysis on the impact of comprehensive state IVF mandates, conducted using 2018 CDC data, found for the first time that live births per cycle were significantly higher in states with comprehensive states mandates (35.4% vs. 33.4%, *p* < .001) [39]. While the mechanism by which comprehensive insurance coverage for IVF leads to improved birth rates remains unclear, it could be that by performing high volumes of cycles in patient populations with historically worse outcomes, clinics in mandated states have developed more effective evidence-based IVF protocols.

#### Patient experience

Insurance coverage through state infertility mandate or private insurance, has a significant impact on the experience of patients seeking fertility treatments. In a recent single institution survey, insurance coverage was the most cited perceived barrier to care [100]. Among those patients who ultimately initiate fertility treatment, financial strain is a commonly cited reason for treatment discontinuation [101]. In a separate single institutional study, women without insurance for IVF were over three times more likely to discontinue IVF treatment after a failed first cycle of IVF. For those who did return, there was a longer time between cycles than for women with insurance [102]. Moreover, in an analysis of over 7,000 respondents to an online questionnaire on the FertilityIQ website, patients reported higher positive experience with their care if they had insurance coverage [103]. These studies indicate that insurance coverage not only improves patient outcomes, but their experience with infertility treatment as well.

### Limitations of state mandates

While insurance mandates are essential to expanding the accessibility of fertility treatments, several significant limitations should be considered. One limiting factor is that mandates only apply to private insurance plans subject to regulation from state governments. Self-insured employers, certain small businesses, and companies with religious objections are often exempt from mandates. Patients that are federally employed or covered under Medicare, Medicaid (with the exception of New York), and TRICARE also do not receive the benefits afforded by state mandates.

Notably, among states with insurance mandates, there is wide variability not only in which services are covered but also in who meets eligibility criteria. Consequentially, entire patient populations who need these services are excluded from coverage (see Table 1). For example, states such as Arkansas, Hawaii, and Texas require that a patient’s eggs be fertilized by her spouse’s sperm to qualify for coverage [33]. An even larger number of states require a specific time period of unprotected intercourse without conception prior to covering fertility treatments [33]. Single individuals, patients in same-sex relationships, transgender patients, and gender nonconforming patients in these states remain vulnerable to disparities in access as a result. Even among heterosexual couples, there is limited support for male factor infertility as approximately half of the current mandates cover the evaluation and treatment of the male partner [104]. Mandates requiring couples to use their own gametes also discriminate against individuals requiring donor eggs, sperm, or embryos, regardless of a potential medical indication necessitating third-party gametes [32, 33, 39]. Furthermore, patients undergoing treatments that may affect future fertility, such as gonadotoxic chemotherapy, are variably included in infertility legislation [34].

Research also indicates that racial and ethnic disparities in access to IVF persist in states with mandates, suggesting that improving insurance coverage for these services is not sufficient for eliminating inequities in infertility care. A study from 2017 using CDC data showed that while states with mandated coverage had overall higher rates of ART utilization compared to states without mandates, black non-Hispanic and Hispanic women still had lower rates of utilization on average [76]. Another recent study showed that black women with insurance were more likely to stop IVF than white women following an initial unsuccessful cycle [102]. Current evidence supports the assertion that income, education, and private insurance mediate the relationship between race, ethnicity, and access to fertility treatments; however, there are additional influential sociocultural and historical factors that are important to consider [73, 105]. Significant work remains to better understand how mandates could more effectively address disparities in fertility care, and hopefully, future health policies will be better equipped to decrease remaining inequities.

## Conclusions

Even though infertility affects men and women equally, women are disproportionately blamed for this condition worldwide and, as such, experience increased stigma and emotional distress associated with the inability to conceive [106]. As women increasingly delay childbearing for a variety of reasons [107, 108], insurance coverage for infertility has emerged as an important means of encouraging educational ambitions, facilitating career opportunities, and promoting gender equality [109]. Alongside existing limitations in access to contraception, abortion, and prenatal care in the US [110–112], fertility treatments are often prohibitively expensive and require not only adequate financial resources, but significant psychosocial support as well. Therefore, infertility care is a fundamental pillar of reproductive health that requires robust cultural and institutional infrastructure to be accessible.

Though we have touched on economic and medical benefits to mandated insurance coverage of infertility, there are strong ethical arguments for these mandates as well. Reproductive justice is defined as the right to maintain bodily autonomy, to have children, to not have children, and to parent children in safe and sustainable environments [113]. The tenants of reproductive justice, coupled with the conceptualization of infertility as a disease, position a lack of coverage for infertility as a critical threat to reproductive autonomy. Moreover, this threat is not felt equally among all patients as those in minority groups and with lower socioeconomic status are disproportionately affected by lack of access [28]. Given that infertility patients with the greatest need suffer from compounding systems of racism, healthcare providers, politicians, researchers, and activists must ensure that their advocacy focuses on the multilayered barriers preventing patients from actualizing their reproductive freedom.

Fortunately, there appears to be popular endorsement of coverage for infertility treatments and ART in the United States. In a recent study of a large, nationally representative sample of US adults, the majority of participants reported supporting insurance coverage for infertility and IVF [114]. US healthcare providers with experience caring for patients with infertility have also demonstrated strong support for coverage of fertility services. In a 2017 SART survey of physicians, embryologists, nurses, and clinic administrators, over 78% of respondents approved of insurance coverage for IVF for individuals requiring infertility treatment [15]. Agreement was nearly unanimous (> 95%) regarding coverage for couples with medical necessity for IVF/PGD, such as carrier couples with the same recessive gene for diseases like cystic fibrosis or sickle cell. Still, significant political gridlock diminishes prospects for federal fertility legislation, meaning the weight of this responsibility rests largely on the shoulders of state governments.

This review highlights the ways in which infertility mandates, specifically those with more comprehensive IVF coverage, result in improvements in the access to and provision of IVF. As more states consider mandates, it is important to recognize their impact in terms of greater per-capita IVF utilization, fewer embryos per transfer, more judicious ICSI and PGT utilization, lower multiple birth rates, and higher live birth rates. While there are limits to the potential impact of state infertility mandates, these laws play a key role in the promotion of evidence-based practices. Importantly, they also represent an essential and impactful strategy for the advancement of reproductive rights.

## Data Availability

Not Applicable.

## Abbreviations

ART: assisted reproductive technology
ASRM: American Society for Reproductive Medicine
CDC: Centers for Disease Control and Prevention
eSET: elective single embryo transfer
HMO: health maintenance organization
ICSI: intracytoplasmic sperm injection
IUI: intrauterine insemination
IVF: in vitro fertilization
OI: ovulation induction
OOP: out-of-pocket
OR: odds ratio
OS: ovarian stimulation
PGT: preimplantation genetic testing
SART: Society for Assisted Reproductive Technology
US: United States
